# Brigadier-General Joseph B. Brown, M. D., U.S. Army

**Published:** 1891-11

**Authors:** 


### Obituary.

Brigadier-General Joseph B. Brown, Surgeon U. S. Army (retired), died Oct. 21, 1891, at his home in Albion, N. Y. Dr. Brown rendered conspicuous service in the late Civil War, and was Medical Director of Keyes's (the fourth), and Franklin's (the sixth) Army Corps during McClellan's peninsular campaign. The writer was Assistant Surgeon of the Forty-ninth Regiment New York Volunteers (General Bidwell's) during this period, and it was Dr. Brown who brought him an order from General Franklin, during the battle of Savage's Station, June 29, 1862, to remain with the wounded that were to be left behind to fall into the enemy's hands. Dr. Brown received his Brevet Brigadier-General for meritorious service during an epidemic of yellow fever several years ago.

